# Radiation-related lymphopenia is associated with spleen irradiation dose during radiotherapy in patients with hepatocellular carcinoma

**DOI:** 10.1186/s13014-017-0824-x

**Published:** 2017-05-30

**Authors:** Jing Liu, Qianqian Zhao, Weiye Deng, Jie Lu, Xiaoqing Xu, Renben Wang, Xia Li, Jinbo Yue

**Affiliations:** 1 Graduate Education Center, Shandong Academy of Medical Sciences, Jinan, China; 2 School of Medicine and Life Sciences, University of Jinan-Shandong Academy of Medical Sciences, Jinan, Shandong China; 3 Department of Radiation Oncology, Shandong Cancer Hospital affiliated to Shandong University Jinan, 440 Jiyan Road, Jinan, Shandong 250117 China; 4 Department of Radiation Oncology, The University of Texas MD Anderson Cancer Center, 1515 Holcombe Blvd, Houston, TX 77030 USA; 5 Division of Epidemiology, Human Genetics and Environmental Sciences, The University of Texas School of Public Health at Houston, 1200 Hermann Pressler St, Houston, TX 77030 USA; 6 Department of Radiation Physics, Shandong Cancer Hospital affiliated to Shandong University, Jinan, Shandong China; 7 Shandong University School of Medicine, Jinan, Shandong 250062 China; 8 Laboratory for TCM Immunopharmacology and Molecular Biology, Institute of Basic Medicine, Shandong Academy of Medical Sciences, 18877 Jingshi Road, Jinan, 250062 China

**Keywords:** Hepatocellular carcinoma, Radiotherapy, Lymphopenia, Spleen dosimetric indicators

## Abstract

**Background:**

The decrease in peripheral blood lymphocytes induced by radiation lessens the antitumour effect of the immune response, which might cause immunosuppression. We aimed to investigate the correlation between the decrease in peripheral blood lymphocytes during radiotherapy (RT) and the spleen irradiation dose in patients with hepatocellular carcinoma (HCC).

**Methods:**

The subjects were 59 patients with HCC who had received RT from 2005 to 2014. The Min ALC (minimum value of absolute counts for peripheral blood lymphocytes) was collected from the routine workup for each patient prior to RT and weekly during RT. Spleen dose-volume variables, including the percentage of the organ volume receiving ≥ n Gy (V_n_) and the mean spleen dose (MSD), were calculated using Eclipse treatment planning. Potential associations between dosimetric variables and the Min ALC were assessed by multiple linear regression analysis.

**Results:**

Peripheral lymphocytes decreased during RT (*P* < 0.001). The Min ALC correlated with the MSD (*P* = 0.005), spleen V_5_ (*P* = 0.001), spleen V_25_ (*P* = 0.026) and spleen V_30_ (*P* = 0.018). Controlling for the Karnofsky performance status (KPS), sex, age, Child-Pugh grade, total dose and tumour stage, a multiple linear regression model with bootstrap analysis of 1000 replicates showed that only the spleen V_5_ was correlated with the decrease in the Min ALC (*P* < 0.05). According to the receiver-operating characteristic (ROC) curve analysis, the predictive cutoff values of the MSD, V_5_, V_25_ and V_30_ of the spleen for the Min ALC were 227.72 cGy, 17.84, 0.98 and 0.42%, respectively (*P* = 0.002, *P* = 0.004, *P* = 0.007 and *P* = 0.002, respectively). Furthermore, an MSD ≥ 227.72 cGy (OR = 14.39; 95% CI, 12.18 to 16.60) and V_5_ (OR = 7.99; 95% CI, 6.91 to 9.07) of the spleen significantly predicted the Min ALC.

**Conclusions:**

Higher spleen irradiation doses were significantly correlated with lower Min ALC during RT for HCC. V_5_ should be limited in clinical practice. Maximum sparing for spleen irradiation during RT is recommended to preserve peripheral blood lymphocytes, which may decrease immunosuppression.

## Background

The immune system plays a crucial role in cancer suppression and determines cancer prognosis [1–3]. Ionizing radiation is one of the mainstay treatments in oncologic diseases, and its effects can lead to apoptosis of circulating lymphocytes, which lessens the antitumour effect of the immune response [4, 5].

Lymphocytes, essential effector cells in antitumour immunity, could specifically recognize and kill tumour cells or release a series of cytokines to activate the host immune system. Accumulating studies have demonstrated that peripheral lymphocytes decrease during the course of radiation therapy (RT) because of their high radiation sensitivity [6]. The minimum value of absolute peripheral lymphocyte counts during RT treatment (Min ALC) has also been considered a prognostic factor for recurrence and survival in several cancers [7–10]. In a previous study, we verified the value of Min ALC in predicting survival in hepatocellular carcinoma (HCC) patients [11]. Many peripheral blood lymphocytes pass through the spleen on a daily basis, and changes in the function of the spleen affects the counts of the lymphocytes in peripheral blood [12]. Meanwhile, the spleen, as a critical component of the immune system, plays an exclusive role in both the innate and adaptive immune systems [13].

Therefore, we hypothesized that a radiation dose to the spleen would reduce the counts of peripheral blood lymphocytes. To test these hypotheses, we investigated the correlation between the decrease in peripheral blood lymphocytes and spleen irradiation dose in patients with HCC during RT.

## Methods

### Patients and therapy

The subjects for this study were 59 patients with inoperable stage II-IV (AJCC; 7th Ed 2010) HCC who received RT at Shandong Cancer Hospital Affiliated to Shandong University between January 2009 and December 2015. All patients satisfied the diagnosis criteria according to radiological or histological findings as recommended by the American Association of the Study of Liver guidelines [14, 15]. Eligibility criteria were as follows: 1) age ≥18 years, 2) Child-Pugh A liver function score, and 3) peripheral white blood cells above 2000 cells/μL during RT treatment without receiving prophylactic or remedial treatment for the decrease in white blood cells during RT. Patients who had a history of prior liver RT or surgery, simultaneous splenectomy or splenomegaly, or hepatitis B virus reactivation during RT were excluded from the study.

All patients received conventional RT with curative intent up to 50–60 Gy and did not receive other anticancer treatments, except for transarterial chemoembolization, after RT. The study was approved by the institutional review board of Shandong Cancer Hospital Affiliated to Shandong University, and all participants gave informed consent to participate.

### Clinical data and assessment of absolute peripheral lymphocytes

Detailed clinical data obtained prior to the initiation of RT were collected from enrolled patients and included patient age, sex, Karnofsky performance status (KPS), blood test results, abdominal enhanced computed tomography (CT) or magnetic resonance imaging (MRI), and American Joint Committee on Cancer (AJCC; 7th Ed 2010) stage. Blood samples were obtained by venous puncture and were collected 0–3 days before the start of RT to quantify the lymphocytes; blood samples were collected again once a week during and after RT. Changes in the lymphocyte counts during RT were evaluated. The Min ALCs during RT treatment were measured. The decrease in lymphocytes was quantified by the difference between the baseline values of the absolute peripheral lymphocyte before RT and the Min ALC during RT. Additionally, we analysed the Min ALC and the days on which the Min ALC were measured.

### Treatment planning and spleen dosimetry

The spleen was contoured for each patient, and its corresponding dosimetry was calculated and approved using the Pinnacle treatment plan by a dosimetrist. Spleen dosimetric variables, including the mean spleen dose (MSD) and a set of volumetric proportions of the spleen receiving ≥ x Gy (V_x_), were extracted from the treatment plan.

### Follow-up

Patients with positive hepatitis B virus DNA were given antiviral drugs (entecavir or lamivudine) throughout the period of antitumour treatment, and no hepatitis virus activation was observed. After RT completion, contrast-enhanced CT scans or MRI of the abdomen were obtained within 3 months and then every 6 months. Overall survival (OS) was calculated from the completion of RT to the date of death from any cause or the last follow-up.

### Statistical analysis

Patient characteristics were evaluated with descriptive statistics. Receiver operating characteristic (ROC) curve analysis was performed to select the most appropriate Min ALC cutoff values for identifying the one-year survival rate and predictive cutoff value of spleen dosimetric variables for the Min ALC. We divided the patients into two groups according to the threshold value of the Min ALC. The lymphocyte cells during RT were compared using Student’s *t*-test. Bivariate correlations were employed to investigate the correlations between the Min ALC value and spleen dosimetric parameters. Spearman correlation coefficients were applied on the association among different dosimetric covariates, then stepwise backward elimination with a selection criterion of *p* < 0.1 was applied to find the best subset of variables. The association between spleen dosimetric characteristics and Min ALC was analysed using a multiple linear regression model. The bootstrapping method was applied for internal validation. External validation could not perform in this study. All statistical analyses were performed using SPSS software (version 20.0; SPSS Inc., Chicago, IL), and a *P* value < 0.05 was considered statistically significant from two-sided tests. The procedures were based on the TRIPOD statement [16].

## Results

### Patient characteristics

The characteristics of the 59 evaluable patients are summarized in Table 1. All patients completed RT without unscheduled interruption. Most patients had a good performance status and stages II-III disease.

**Table 1:** Patient and treatment characteristics

Characteristic	n (%) or median (range)
Sex	
Male	46 (82.14%)
Female	10 (17.86%)
Age (years)	61 (34–84)
Karnofsky performance status (10%)	
≤ 80	34 (60.71%)
> 90	22 (39.29%)
Hepatitis B virus	
Positive	49 (87.5%)
Negative	7 (12.5%)
Stage^a^	
II	13 (23.21%)
III	25 (44.64%)
IV	18 (32.14%)
Treatment strategy	
RT alone	9 (16.07%)
RT combined with TACE	47 (83.93%)
Prescription dose for liver tumor (cGy)	5400 (4500–6200)
Live tumor(cm^3^)	151.81 (4.20–976.93)
Plan target volume for liver tumor(cm^3^)	342.29 (19.20–1442.27)

*V*_*5*_ volumetric proportion of spleen receiving ≥ 5 Gy

^a^American Joint Committee on Cancer staging manual, seventh edition. Frequency (percent proportions) are shown for all categorical variables and Medians (range) are shown for all continuous variables

### Predictive cutoff value of the Min ALC to survival

**Fig. 1:**
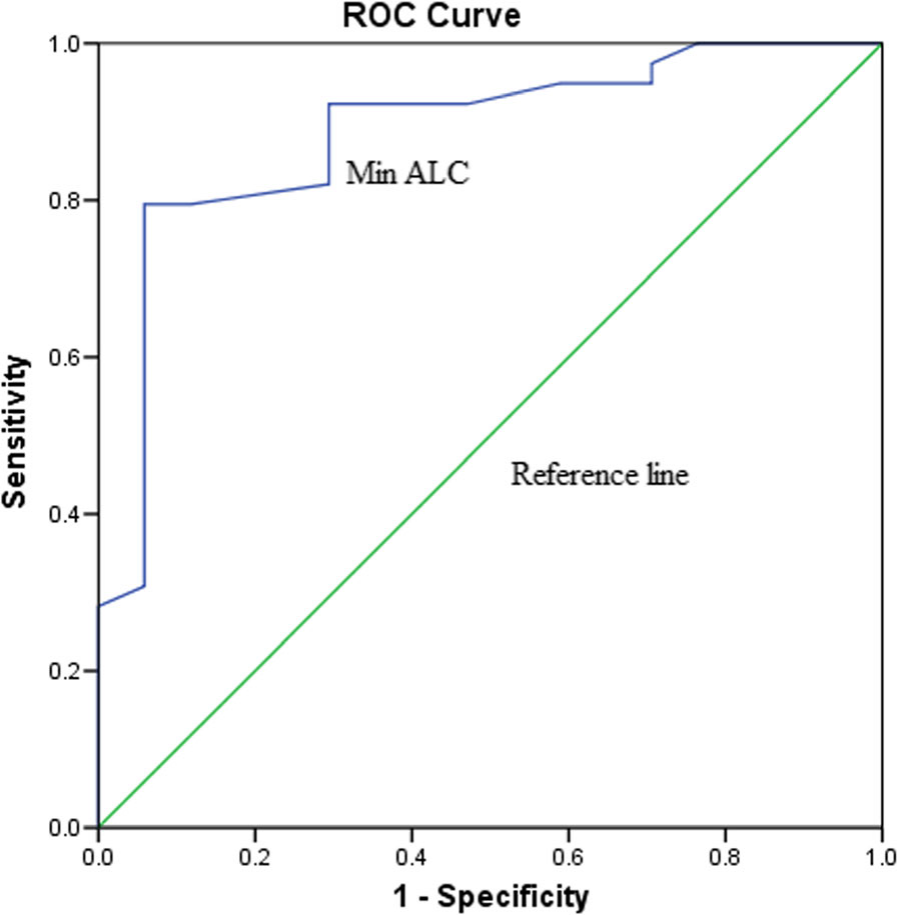
Determination of the cutoff value for the minimum absolute lymphocytes (Min ALC) in predicting the one-year survival in HCC patients who undergo radiotherapy

At the end of the follow-up period, 19 (33.93%) patients were alive. The 1-year OS rate was 22.03%. ROC curve analysis identified an optimal cutoff point of 300 cells/μL for the Min ALC to predict the 1-year OS [odds ratio (OR) = 28.8; 95% (Confidence interval) CI = 27.23–30.37; Fig. 1]. The sensitivity and specificity of the Min ALC for predicting one-year survival rates were 92 and 71%, respectively [*P* < 0.001 and area under curve (AUC) = 0.885]. The baseline characteristics of patients with respect to the cutoff Min ALC of 300 cells/μL are shown in Table 2. No significant differences were found between the two groups. In addition, no correlation was found between spleen dosimetric variables and survival (all *P* < 0.05).

**Table 2:** Relationships of and clinical characteristics Min ALCs in patients with hepatocellular carcinoma

Variables	Min ALCs ≤ 300 (n = 15)	Min ALCs > 300 (*n* = 41)	*P* value
Age (years, median, range)	57 (34–84)	60 (46–79)	0.239
Gender (male/female)	13/2	33/8	0.593
KPS (>90/≤80)	6/9	13/28	0.562
Hepatitis B virus (+)/(−)	13/2	36/5	0.909
Stage^a^			
II	1	12	
III or IV	14	29	0.076

*Min ALC* minimum absolute lymphocyte counts during radiotherapy

^a^American Joint Committee on Cancer staging manual, seventh edition. Frequency (percent proportions) are shown for all categorical variables and Medians (range) are shown for all continuous variables

### Correlations between Min ALC and spleen dosimetric variables

**Fig. 2:**
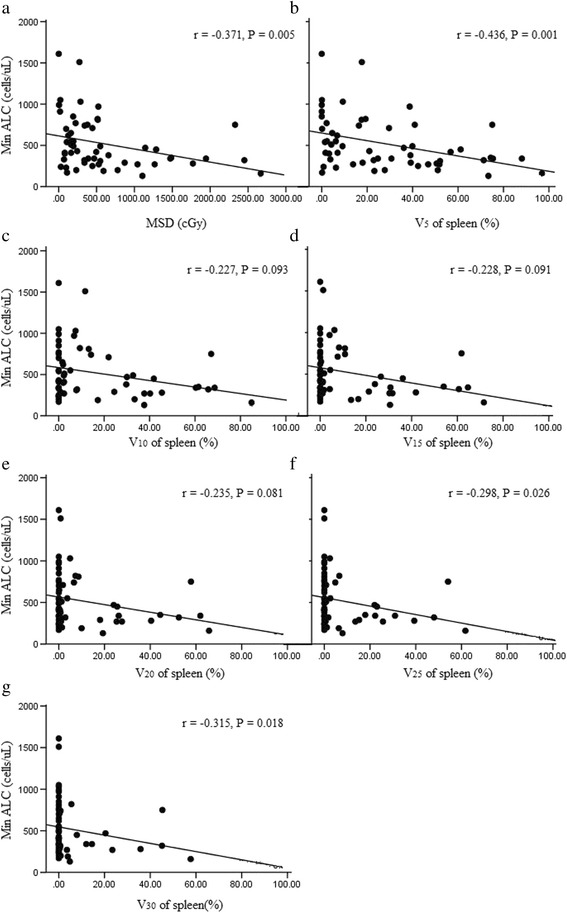
Correlation between the peripheral minimum absolute lymphocytes (Min ALC) during radiotherapy treatment with the mean dose of spleen (MSD) (**a**), spleen V_5_ (the percentage of spleen volume receiving ≥ 5 Gy) (**b**), V_10_ (**c**), V_15_ (**d**), V_20_ (**e**), V_25_ (**f**) and V_30_ (**g**). Spearman correlation coefficients (r) and corresponding *P* values are shown

The peripheral blood lymphocytes decreased during RT (1557.50 ± 574.19 vs. 520.89 ± 318.82 cells/μL; *P* < 0.001). Spearman correlation analysis revealed that spleen dosimetric variables, including the MSD, V_5_, V_25_ and V_30_, were significant factors correlated with the decrease in lymphocytes (*P* = 0.005, 0.001, 0.026, and 0.018; Fig. 2). V_10_, V_15_ and V_20_ of the spleen were not found to be statistically significant factors (*P* = 0.093, 0.091 and 0.081, respectively; Fig. 2). Spearman correlation also revealed that there were strong associations among different dosimetric variables (all *P* < 0.05). Among them, V_5_ was selected as the best fitting model by stepwise selection. In multiple linear regression, when controlling for patients’ KPS, sex, age, Child-Pugh grades, total dose and tumour stage, only V_5_ was demonstrated to be an independent predictor for a decrease in lymphocytes (beta coefficient = −3.523; 95% CI, −6.554 to 0.100; *P* = 0.034).

### Predictive cutoff value of spleen dosimetric variables for the Min ALC

**Fig. 3:**
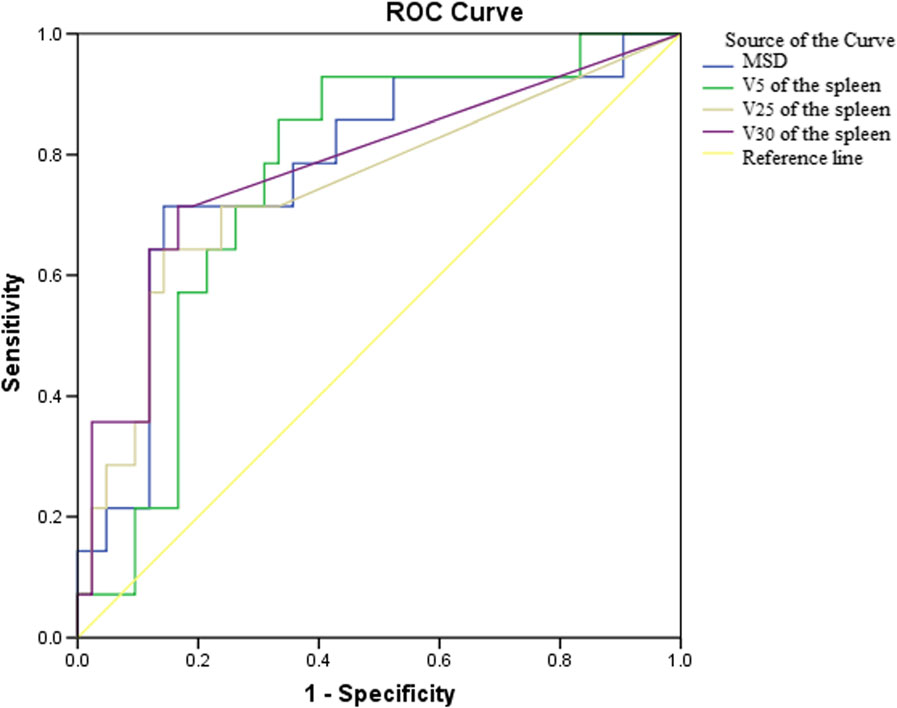
ROC curve analysis for determining the cutoff value of spleen dosimetric variables in predicting the minimum absolute lymphocytes (Min ALC)

ROC curve analysis was performed to determine the predictive accuracy of the spleen dosimetric variables (MSD, V_5_, V_25_ and V_30_ of the spleen) for the Min ALC. According to the ROC curve analysis, the predictive cutoff values of the MSD, V_5_, V_25_ and V_30_ of the spleen for the Min ALC were 227.72 cGy, 17.84, 0.98 and 0.42%, respectively (*P* = 0.002, *P* = 0.004, *P* = 0.007 and *P* = 0.002, respectively; Fig. 3.). Using these cutoff values, an MSD ≥ 227.72 cGy (OR = 14.39; 95% CI, 12.18 to 16.60) and V_5_ (OR = 7.99; 95% CI, 6.91 to 9.07) of the spleen significantly predicted the Min ALC (Table 3).

**Table 3:** Predictive cutoff value of spleen dosimetric variables and its determining ability to Min ALC

Parameters	Threshold	Accuracy	OR	95% CI
MSD	227.72 cGy	77.70%	14.39	12.18 to 16.60
V_5_	17.84%	75.90%	7.99	6.91 to 9.07
V_25_	0.98%	74.30%	0.77	0.08 to 1.46
V_30_	0.42%	77.70%	0.53	−0.13 to 1.19

*MSD* mean dose of spleen, *V*_*x*_ volumetric proportion of spleen receiving ≥ x Gy, *Min ALC* minimum absolute lymphocyte counts during radiotherapy, *OR* odds ratio, *CI* confidence interval

## Discussion

In this study, we observed that a higher Min ALC after RT (with a cutoff value at ≥ 300 cells/μL) predicts better 1-year overall survival based on 59 HCC patients. We further found that spleen irradiation dose was significantly correlated with a lower Min ALC during RT. Therefore, the maximum sparing for spleen irradiation during RT is recommended to preserve peripheral blood lymphocytes, which may decrease immunosuppression. Furthermore, we identified the predictive cutoff value of spleen dosimetric variables for the Min ALC to identify the spleen dosimetric constraints for clinical practice.

The occurrence and progression of a tumour result from the interplay between the tumour-promoting environment and host immune system [17]. Lymphocytes, as one of the main types of immune cells, play a crucial role in antitumour immunity and affect cancer development and progression [6, 8, 18]. Lymphocyte-mediated antitumour immunity is not only involved in carcinogenesis, progression and recurrence of carcinoma, but also in the clinical response and follow-up to treatment [17]. Nevertheless, lymphocytes are categorized as radiation-sensitive cells and can be induced to undergo apoptosis with irradiation [9, 19], which may compromise antitumour immunity. Other studies have shown that lymphopenia is a surrogate marker for the level of immunosuppression and an effective marker for the prognosis of several cancers [4, 20, 21]. In a previous study, we demonstrated that Min ALCs during RT have an independent predictive value for the overall survival in HCC patients [11]. In the current study, we further identified the optimal cutoff value of Min ALC during RT in predicting one-year survival. The underlying mechanism for our findings is that immunosuppression may lead to a high risk of tumour growth, relapse and metastasis [22–24]. Our finding could be useful for identifying patients who have a high risk of a decreased peripheral lymphocyte count during antitumour treatment and who may need immune therapy or more intensive follow-up for tumour progression. Further phenotypic and functional analyses of the lymphocyte subset during RT would help clarify the mechanisms underlying the responsiveness of tumours to RT.

The spleen is the largest peripheral immune organ, and it plays important roles in regulating immune responses. The specialized structure of the venous system of the red pulp gives it a unique capacity to filter blood. Different cell components in the blood, including a large number of lymphocytes, pass through the spleen on a daily basis [13, 25]. Our study identified a significant correlation between the spleen irradiation dose and a decrease in peripheral lymphocytes during RT based on the trend that patients with higher spleen radiation exposure have a lower Min ALC during RT.

The spleen is not routinely treated as an at-risk organ in treatment planning, and the tolerance of the spleen to radiation has yet to be fully established. In the current study, the only significant independent parameter associated with the Min ALC, as determined by multivariate analysis, was the spleen V_5_. Therefore, it appears that the low-dose area plays a major role in decreasing lymphocyte counts. Reducing the spleen low-dose area may be critical to reducing the risk of a decrease in the peripheral lymphocyte counts. To the best of our knowledge, this study is the first to explore the correlation between spleen irradiation and peripheral lymphocytes during RT and to further identify the spleen dosimetric constraints used for clinical practice. Our results suggest that we should try to decrease spleen irradiation, which may prevent radiation-induced lymphopenia and improve the overall survival for HCC patients.

This study has several limitations. First, it is a retrospective analysis that is subject to all the limitations of post hoc analyses. Second, our sample size is relatively small. Third, our work is the lack of any external validation of these data. This fact, with the low number of cases, could increase the value of the overfitting and returning higher significance level than expected with higher case numbers. Prospective studies are warranted to validate our findings.

## Conclusions

In summary, our study is the first to demonstrate a dose-response correlation between a higher spleen irradiation dose and lower Min ALC. This finding indicates that the spleen should be treated as an at-risk organ and supports the need to identify possible dose constraints for HCC patients undergoing RT treatment. Prospective studies assessing changes in the peripheral lymphocytes in patients who have undergone spleen sparing during RT may help better define constraints for this organ in the future.

## Abbreviations

AJCC: American Joint Committee on Cancer
AUC: Area under curve
HR: Hazard ratio
CT: Computed tomography
HCC: Hepatocellular carcinoma
KPS: Karnofsky performance status
Min ALC: Minimum lymphocyte cell
MRI: Magnetic resonance imaging
MSD: Mean spleen dose
OS: Overall survival
ROC: Receiver-operating characteristic
RT: Radiation therapy
TACE: Transarterial chemoembolization
V_x_: Volumetric proportion of spleen receiving ≥ x Gy
